# Dark chocolate and reduced snack consumption in mildly hypertensive adults: an intervention study

**DOI:** 10.1186/s12937-015-0075-3

**Published:** 2015-08-22

**Authors:** Raika Koli, Klaus Köhler, Elina Tonteri, Juha Peltonen, Heikki Tikkanen, Mikael Fogelholm

**Affiliations:** 1 Nutrition, Department of Food and Environmental Sciences, University of Helsinki, P.O. BOX 66, FI-00014 Helsinki, Finland; 2 Department of Sports and Exercise Medicine, Institute of Clinical Medicine, Biomedicum Helsinki, University of Helsinki, P.O. Box 20, FI-00014 Helsinki, Finland; 3 Institute of Biomedicine, University of Eastern Finland, Kuopio Campus, P.O. Box 1627, FI-70211 Kuopio, Finland

## Abstract

**Background:**

Several studies have shown that cocoa and cocoa-containing foods have the potential to lower blood pressure and improve endothelial function. Most of the studies reporting the beneficial effects of dark chocolate on blood pressure have been short (≤ 4 weeks). The aim of the present 8-wks (weeks) study was to assess the effects of regular consumption of dark chocolate during a reduced snack consumption intervention on blood pressure and other cardiovascular risk factors in mildly hypertensive individuals.

**Design:**

This was a randomized, controlled, cross-over trial involving 22 adults (8 women, 14 men), aged 33–64 y, BMI 27.7 ± 3.7 kg/m^2^ with mild hypertension. During the intervention period (8-wks) the participants reduced the intake of habitual snacks and replaced them with dark chocolate (49 g/day). In the control period, they only reduced the snacks without any added chocolate. Data (blood lipid profile, glucose, insulin, 24 h blood pressure) was collected in the beginning and end of both periods (intervention and control), and some variables also in the run-in and run-out periods (weight, body fat percentage, blood pressure, arterial stiffness index, diet and physical activity).

**Results:**

Daily consumption of dark chocolate had no effects on 24 h blood pressure, resting blood pressure (mean ± SD, pre 142 ± 11.5/89 ± 8.4 mmHg vs. post 142 ± 14.2/88 ± 9.4 mmHg in systolic and diastolic blood pressure, respectively) or arterial stiffness (mean ± SD, pre 7.68 ± 0.88 vs. post 7.76 ± 0.89).

Weight was reduced by 1.0 ± 2.2 kg during the control (reduced snack only) period, but was unchanged while eating chocolate (p < 0.027 between the treatments).

**Conclusion:**

The data collected in this study indicates that inclusion of dark chocolate daily in the diet had no significant effects on blood pressure or other cardiovascular risk factors during a reduced snack period.

**Trial registration:**

ClinicalTrials.gov identifier NCT02130141

## Background

Polyphenols are plant-derived compounds; secondary metabolites with an essential role in protection of plants against different environmental and microbial threats [1]. Polyphenols can be divided into flavonoids and non-flavonoids. Flavonoids consist of six subgroups, namely flavonols, flavan-3-ols (monomeric and polymeric structures), flavones, isoflavones, flavanones, and anthocyanidins. Phenolic acids, stilbenes, lignans and other polyphenols belong to non-flavonoid group.

Unlike vitamins, polyphenols are not essential nutrients. However, regular intake of polyphenols may have favorable effects on health and risk of chronic diseases. Also, several clinical trials have shown a favorable effect of flavonoids on cardiovascular disease (CVD) risk factors [2, 3]. The health effects of polyphenols depend on their bioavailability [4], e.g., the rate and extent of their absorption and metabolism, and the chemical structure of the molecule.

Cocoa and cocoa-containing foods, such as dark chocolate, represent a very rich source of flavonoids as they provide a higher content of flavonoids per serving than red wine or tea [5]. Cocoa contains monomeric flavanols, namely epicatechin and catechin, and oligomeric procyanidins [6]. The consumption of cocoa products has been shown to have an influence on CVD risk factors [2, 7], and cocoa intake has been inversely associated with cardiovascular mortality [8]. Epidemiological studies have also reported an inverse association between chocolate consumption and risk of CVD [9] and heart failure [10]. In human intervention studies dark chocolate consumption has shown health promoting effects on blood pressure [11, 12], total-, LDL- and HDL-cholesterol [11, 13], and also on insulin resistance and sensitivity [11].

The beneficial health effects of cocoa have been especially related to endothelial function [14]. Consumption of cocoa and chocolate has been shown to decrease blood pressure and to ameliorate flow mediated dilation (FMD) [7]. Moreover, isoflavones, anthocyanins and cocoa flavan-3-ols in particular, have been associated with or have been shown to have a positive effect on arterial stiffness [15].

Most of the studies reporting the beneficial effects of dark chocolate on blood pressure have been short (i.e., 2 to 4 wks) and the daily dose substantial, as much as 100 g/day [16–18]. Therefore, in this study we wanted to find out if a more reasonable portion (49 g/day dose) for a longer period of time would have an effect on blood pressure and arterial stiffness. The aim of the present cross-over study was to examine the effects of daily consumption of dark chocolate during a reduced snack consumption period for 8 wks on blood pressure (primary outcome) and other cardiovascular risk factors in adults with mild hypertension. For ethical reasons, we wanted to avoid deliberate positive energy balance in our participants with an increased risk for chronic diseases. Therefore, during both the intervention (dark chocolate) and control (no chocolate) periods, the energy intake in the habitual diet was reduced by ways of reducing the participant’s daily snack consumption.

## Methods

### Participants

Thirty healthy volunteers (19 men, 11 women) were recruited by flyers and newspaper advertisements. To be included into the study, the participants had to have mild hypertension (140–159 mmHg systolic blood pressure (SBP) or 90–99 mmHg diastolic blood pressure (DBP) according to the Finnish Hypertension Society) and BMI < 35 kg/m^2^. Exclusion criteria were smoking and regular use of medications for cardiovascular disease, diabetes or asthma. Twenty two participants (14 men and 8 women), 33–64 years old, mean age 45.8 (SD 8.3) years with a BMI of 27.9 (SD 3.6) kg/m^2^ completed the study and were included in the final analysis. Eight participants withdrew during the study for personal reasons or because their blood pressure exceeded the accepted level (159/99 mmHg). Written informed consent was obtained from all participants. The study protocol was reviewed by the Ethics Committee of the Helsinki and Uusimaa Hospital District, Finland. The study was registered at the US National Institutes of Health clinical trials database (ClinicalTrials.gov), identifier NCT02130141.

### Study design

**Fig. 1:**
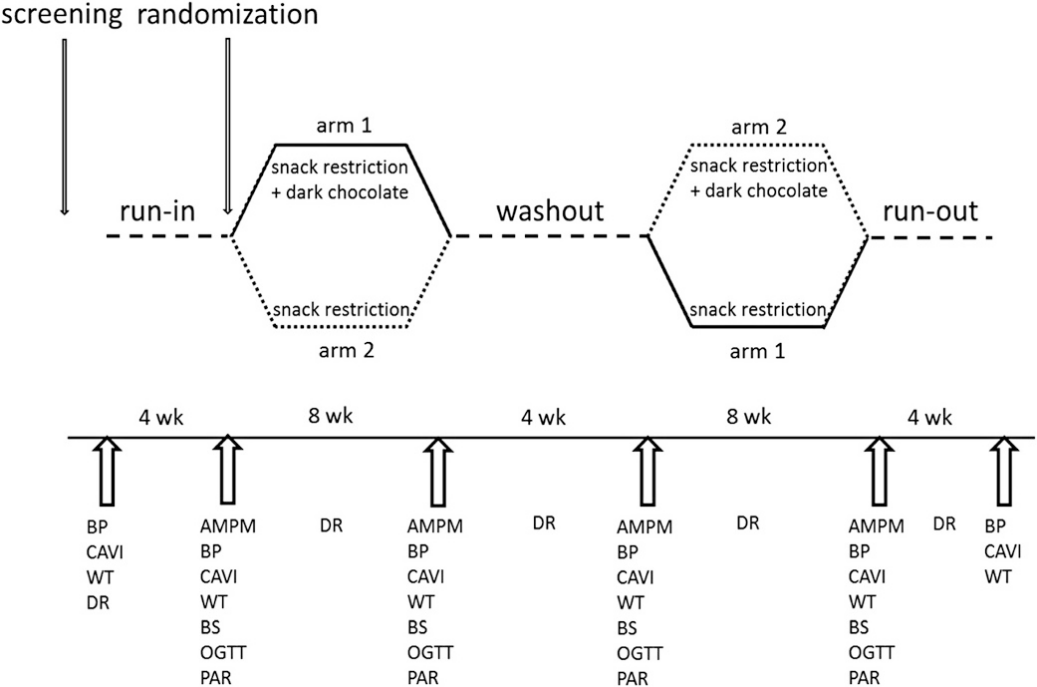
Study design and data collection. After a cocoa free run-in phase of 4 weeks, participants were randomly assigned to follow the snack restriction or receive 49 g dark chocolate in addition to snack restriction. Successively, participants entered a further cocoa-free washout phase of 4 weeks and then were crossed over to the other treatment. The final phase was the run-out period of 4 weeks. Abbreviations: BP = clinical blood pressure; CAVI = cardio-ankle vascular index; WT = weight and other; DR = diet records; anthropometrics; AMPM = 24-h ambulatory blood pressure; BS = blood samples; OGTT = oral glucose tolerance test; PAR = physical activity records

The study was a randomized, controlled, 8-wks cross-over intervention with two arms (Fig. 1). The participants were randomly assigned to one of the two arms (denoting order of interventions) after stratification by sex and BMI. The run-in phase before, and the run-out phase after the intervention and control periods, as well as the wash-out period between them, were all four wks in duration. The participants were asked to maintain their normal dietary and lifestyle habits throughout the study except for the snack restriction. During both the intervention and the control periods, the participants were instructed to reduce their habitual daily snacking, with an aim to reduce energy intake by 250 kcal/day. During the dark chocolate period (8 wks) the participants replaced their habitual snacks with dark chocolate (49 g/day, equals 262 kcal). During the control period, reduced snack consumption was not replaced by dark chocolate. The reduction of energy intake due to reduced snack consumption was planned individually, together with a nutritionist, and based on self-reported food intake and individual preferences. The compliance with the snack restriction was evaluated at personal meetings at the halfway of both intervention periods.

The intervention chocolate was Karl Fazer’s dark chocolate (70 % cacao). The participants were asked to eat seven pieces of the chocolate (7 g) daily, for a daily intake of 49 g/day. The analyzed content of proanthocyanidins (referred as flavanols, by degree of polymerization 1–10) of dark chocolate was 1230 mg/100 g (fresh weight) [19]. The dark chocolate contained total fat 41 g, protein 9 g, carbohydrate 35 g, fibre 11 g and sodium 46 mg per 100 g fresh weight. All the dark chocolate was provided at the first visit of the chocolate treatment period. The daily timing for the chocolate snack was free. White chocolate was allowed throughout the study, but milk chocolate was not.

## Methods

Blood samples were taken and 24 h blood pressure was measured in the beginning and end of both intervention periods. Additionally, blood pressure, weight and anthropometrics, as well as diet and physical activity, were assessed also in the beginning of the run-in period and in the end of run-out period. These visits were also for compliance checks, meetings with the study nurse, and dietary counselling.

### Blood pressure and cardio-ankle vascular index (CAVI)

Ambulatory 24 h blood pressure (systolic and diastolic blood pressure of 24 h average, day time and night time separately) was monitored on a day of standard physical activity, with an adequate cuff for the size of the patient’s arm. Welch Allyn ABPM 6100 (Welch Allyn Inc, USA) validated according to the protocol of the Finnish Hypertension Society, was used. The recorder was programed to take blood pressure measurements every 20 min during daytime and every 45 min during nighttime. The data were considered valid when ≥ 80 % of all measurements were acceptable. Participants kept a brief diary to record timing of activities, sleep and posture when measuring blood pressure.

Cardio-Ankle Vascular Index (CAVI) was measured by VaSera (Fukuda Denshi Co., Ltd., Tokyo, Japan). CAVI is an index reflecting the stiffness of the artery from the heart to ankles. The calculation of CAVI is based on the stiffness parameter β which is measured by carotid echography and is not affected by blood pressure [20]. The echography acquires an oscillometric method for blood pressure measurement, and hence it indicates the natural vascular stiffness. In addition, it measures blood pressure from all four limbs, at first from the right brachial and ankle and then from the left brachial and ankle. Thus, arteries at the right and left sides are alternately pressurized while the other side remains open.

### Weight and body composition

Weight and body composition were measured by body composition analysis device InBody720 (InBody 720, Body Composition Analyzer, Biospace Co. Ltd) with participants in underwear, in a fasted state, and within 20 min after bladder emptying.

### Blood samples and oral glucose tolerance test

Blood samples were drawn after a 10–12 h overnight fast in the beginning and after the 8 wk intervention periods. Blood samples were collected for determination of lipids (total cholesterol, LDL cholesterol, HDL cholesterol, triglycerides), glucose and insulin. Serum (without anticoagulant) and plasma (EDTA-coated tubes) were separated by centrifugation (10 min at 3000 rpm) and frozen at −20 °C until analysis. In addition, an oral glucose tolerance test (OGTT) with 75 g of glucose in 300 ml water was administered to all participants before and after the intervention periods.

Total cholesterol, triglyceride, HDL- and LDL cholesterol concentration were measured enzymatically. Glucose was measured by the hexokinase method and insulin was assayed by the chemiluminescent immunoassay method. To assess glucose response and total insulin secretion, areas under the response curve to glucose and insulin (Glu_AUC_, Ins_AUC_) were calculated from 0 (fasting), 15, 30, 45, 60, 90 and 120 min serum glucose and insulin concentrations using the trapezoid rule [21]. Homeostasis model assessment of insulin resistance (HOMA) was used as a simple index for insulin sensitivity. HOMA was calculated by the formula: HOMA = fasting serum insulin (mU/l) × fasting serum glucose (mmol/L)/22.5 [22].

### Dietary analyses and other confounders

Dietary intake was repeatedly evaluated to ensure the success of the snack restriction and to give an indication of other dietary components related to the outcome variables. The participants completed a 3-day food diary (2 working days and 1 weekend day) five times during the study (the intervention periods, the run-in, wash out and run-out periods). The consumption of all foods and drinks was recorded during these days. Household measures and standard units were used to describe amounts of foods consumed. The volunteers received detailed instructions on how to fill in the diaries, and a nutritionist performed an interview to review and clarify the food records. Food and nutrient intakes were calculated by using AivoDiet software (version 2.0.2.3.; Aivo Finland Oy, Turku, Finland), which is based on Fineli, the Finnish food composition database (The National Institute for Health and Welfare, Helsinki, Finland, 2014). According to self-report, all participants consumed the requested daily amount of dark chocolate. Additionally, the participants refrained from consuming cocoa or chocolate from sources other than the study products during the dark chocolate intervention and control periods. Moreover, the participants did not consume any milk or dark chocolate during the run-in, run-out, or wash-out periods.

Participants were asked to maintain their usual level of physical activity during the study. Additionally, participants’ physical activity and health status, use of medication, as well any adverse events, were evaluated by a questionnaire concerning the previous week before the data collection.

### Statistics and power calculation

Statistical analyses were performed by using IBM SPSS Statistics for Windows (version 21.0; IBM, New York, NY). The normality of the analyzed variables was tested using Shapiro-Wilk’s W-test. If the distribution was not normal, logarithmic transformations were done before the analyses. The differences for the variables between two arms at the baseline were tested with Student’s *t* test for independent samples. Differences in the variables measured between the chocolate and control periods were analyzed with repeated measures ANCOVA, and baseline value as covariate were used. The power calculation was based on data from the meta-analysis by Reid et al. [23]. The required number of participants was 23, when using a cross-over design, with an expected mean difference of 2.8 (expected SD 4.6) mmHg in systolic blood pressure (α- risk = 0.05, power =0.80).

## Results

There were no significant differences in measured variables between the two arms at baseline. Neither 24 h nor clinical blood pressure was significantly affected by the dark chocolate intervention. However, blood pressure decreased significantly over the entire study. Twelve of the 22 participants’ status improved from hypertensive to normotensive. From baseline to the end of the study, mean (SD) systolic and diastolic blood pressures among all participants were reduced by 7 mmHg (12.2) and 3 mmHg (7.9), respectively (p = 0.016, data not shown). The intervention trial had no effect on CAVI.

Weight decreased 1.0 kg (± SD 2.2) after the control period where no dark chocolate was consumed (Table 1). In contrast, there was no decrease in weight during the dark chocolate intervention. The mean difference in weight change between the periods was 1.0 kg (p = 0.027). The percentage of body fat and fat mass tended to decrease during both chocolate intervention and control periods, but the changes were not significant. Other anthropometric measures remained unchanged. Moreover, there were no significant changes or differences in lipid profile, blood glucose or blood insulin during the trial or between the intervention and control periods (Table 2).

**Table 1:** Blood pressure and anthropometrics before and after the 8-wk intervention periods

		Dark chocolate period		Control period		
		baseline^a^	8 wk	baseline	8 wk	*p* ^b^
clinical SBP^c^	(mmHg)	142 (11.5)	142 (14.2)	142 (11.7)	141 (9.9)	*ns.*
clinical DBP^d^	(mmHg)	89 (8.4)	88 (9.4)	89 (9.0)	88 (10.1)	*ns.*
24 h SBP (24 h)^e^	(mmHg)	137 (11.4)	138 (9.9)	140 (9.8)	142 (10.5)	*ns.*
24 h DBP (24 h)	(mmHg)	82 (10.2)	82 (9.2)	84 (9.4)	84 (9.6)	*ns.*
day SBP (24 h)	(mmHg)	142 (11.2)	143 (9.2)	144 (7.8)	146 (10.6)	*ns.*
day DBP(24 h)	(mmHg)	86 (10.2)	86 (8.8)	87 (9.0)	88 (10.1)	*ns.*
night SBP(24 h)	(mmHg)	120 (11.4)	120 (13.3)	121 (10.1)	125 (11.8)	*ns.*
night DBP(24 h)	(mmHg)	69 (10.1)	67 (10.4)	68 (10.6)	70 (8.4)	*ns.*
night SBP dip^f^	(%)	15.1 (3.7)	16.4 (5.7)	15.8 (3.9)	14.3 (5.8)	*ns.*
night DBP dip	(%)	20.4 (5.1)	21.6 (6.2)	21.9 (6.9)	20.5 (6.5)	*ns.*
CAVI^g^ (right)		7.68 (0.88)	7.76 (0.89)	7.92 (0.98)	7.87 (0.90)	*ns.*
CAVI (left)		7.63 (0.88)	7.60 (0.88)	7.74 (0.94)	7.77 (0.90)	*ns.*
weight	(kg)	85.5 (16.4)	85.4 (16.0)	85.7 (16.5)	84.7 (16.4)	*0.027*
fat mass	(kg)	24.4 (10.3)	23.7 (10.2)	24.3 (9.9)	23.5 (9.8)	*ns.*
body fat	(%)	28.0 (9.0)	27.2 (9.2)	27.8 (8.6)	27.3 (9.1)	*ns.*
visceral fat	(mm^2^)	125 (34)	123 (35)	126 (34)	121 (33)	*ns.*

^a^Data are given as mean ± SD; ^b^test the change difference between two periods; ^c^systolic blood pressure; ^d^diastolic blood pressure; ^e^24 h ambulatory blood pressure; ^f^proportional dip from day time (awake) average BP to night time (sleeping) average BP; ^g^cardio-ankle vascular index

**Table 2:** Serum glucose, insulin and lipids before and after the 8-wk intervention periods

		Dark chocolate period		Control period		
		baseline^a^	8 wk	baseline	8 wk	*p* ^b^
glucose	(mmol/L)	5.3 (0.4)	5.4 (0.3)	5.2 (0.4)	5.4 (0.4)	*ns.*
insulin	(mU/L)	8.9 (4.6)	8.6 (3.0)	9.4 (3.5)	9.4 (3.7)	*ns.*
HOMA index^c^		2.12 (1.17)	2.06 (0.71)	2.09 (1.00)	2.27 (0.96)	*ns.*
GLU_AUC_^d^		14.8 (2.8)	14.7 (2.4)	14.3 (2.6)	14.5 (2.5)	*ns.*
INS_AUC_^d^		114.4 (71.0)	112.8 (57.7)	110.8 (73.7)	110.4 (64.4)	*ns.*
total cholesterol	(mmol/L)	5.6 (1.1)	5.6 (1.1)	5.6 (1.1)	5.4 (1.1)	*ns.*
LDL- cholesterol	(mmol/L)	3.6 (1.0)	3.6 (1.0)	3.6 (0.9)	3.4 (1.0)	*ns.*
HDL- cholesterol	(mmol/L)	1.5 (0.3)	1.6 (0.3)	1.5 (0.3)	1.6 (0.3)	*ns.*
triglyserides	(mmol/L)	1.23 (0.47)	1.19 (0.50)	1.27 (0.42)	1.16 (0.48)	*ns.*

^a^Data are given as mean ± SD; ^b^test the change difference between two periods; ^c^the homeostasis model assessment index; ^d^areas under the response curve to glucose and insulin were calculated from the fasting, 15, 30, 45, 60, 90 and 120 min serum glucose and insulin concentrations using the trapezoid rule

According to the 3-d food records, calcium intake was higher during the control period than during the dark chocolate period (Table 3). There were no other significant differences in nutrient intake between the two periods. Physical activity neither changed during the study nor differed between the intervention and control period of the study. The average time spent on leisure time, moderate or vigorous exercise, was on average 28 min per day (data not shown).

**Table 3:** Mean daily intake of selected nutrients according to the 3-d food records

		Chocolate period		Control period		
		baseline^a^	8 wk	baseline	8 wk	*p* ^b^
Energy	(kcal)	2023 (623)	2137 (632)	2068 (505)	2214 (656)	*ns.*
Carbohydrates	(g)	217 (93)	238 (93)	226 (69)	241 (82)	*ns.*
Protein	(g)	87 (20)	87 (29)	85 (24)	97 (37)	*ns.*
Fat	(g)	77 (28)	80 (27)	81 (22)	80 (32)	*ns.*
saturated fat	(g)	29.5 (12.1)	29.3 (10.6)	32.0 (11.5)	28.6 (12.2)	*ns.*
monounsaturated fat	(g)	26.7 (10.8)	28.7 (10.1)	26.6 (7.7)	27.6 (11.6)	*ns.*
polyunsaturated fat	(g)	11.7 (4.4)	12.8 (5.2)	12.1 (3.3)	12.2 (6.5)	*ns.*
Fiber	(g)	20.2 (4.6)	22.3 (8.4)	22.4 (8.9)	20.6 (6.4)	*ns.*
Vitamin C	(mg)	132 (60)	136 (61)	94.5 (72)	134 (94)	*ns.*
Vitamin D	(μg)	8.0 (5.1)	8.2 (4.7)	8.8 (4.3)	10.5 (9.7)	*ns.*
Calcium	(mg)	1061 (457)	1061 (493)	1036 (337)	1153 (412)	*0.019*
Magnesium	(mg)	364 (89)	400 (115)	397 (113)	398 (112)	*ns.*
Sodium	(g)	2.9 (0.89)	2.9 (0.88)	3.0 (0.72)	3.0 (0.97)	*ns.*
Potassium	(g)	4.0 (1.0)	4.1 (1.1)	3.6 (1.3)	4.1 (1.2)	*ns.*

^a^Data are given as mean ± SD; ^b^test the change difference between two periods

## Discussion

In this study, participants limited their habitual snack consumption twice for an 8-wk period. During one of these periods, the snacks were replaced with dark chocolate. As a consequence of snack restriction, the participants achieved a small weight loss, which was restrained by the dark chocolate consumption. Apart from that, the study found neither harmful, nor positive, health effects of chocolate on CVD risk factors in mildy hypertensive participants.

Meta-analyses have concluded that cocoa-rich products may reduce blood pressure [23]. However, there is no clear separation between chocolate, and cocoa or flavan-3-ols as such, and conclusions are inferred from the above-mentioned compounds together. Another challenge in randomized, controlled trials, is which compound or product should be used as the control. If the aim is to have two treatments with equal energy intake, a control product is required.

In the present study, we investigated the effect of dark chocolate on blood pressure without using control products. In previous studies, where dark chocolate was consumed without a control product [12, 13, 24–26], or with a lycopene capsule [27] as the “control” treatment, only Almoosawi et al. [24] and Desch et al. [12] found a significant effect on blood pressure. In these studies, participants received 20 g polyphenol-rich dark chocolate with 500 and 1000 mg polyphenols for 2 weeks, and either 6 or 25 g/day of flavanol-rich dark chocolate for 3 months, respectively. Researchers proposed that the observed effects of polyphenol –rich dark chocolate were linked to improved cortisol metabolism in their overweight and obese participants [24]. In the latter study, all except one patient received at least one antihypertensive agent, hence the possible influence of medications couldn’t be excluded. Also, as the authors mentioned, they could not ignore that, apart from a true antihypertensive effect of dark chocolate, regression to the mean might also have been responsible for the observed BP reductions [12].

All other studies [11, 16–18, 28–31], which reported the ability of dark chocolate to lower blood pressure, used white chocolate, cocoa butter or chocolate bars without flavanols as a control, in order to create conditions with equal energy intake. The lack of control product is a limitation of our study. However, it is not clear whether these control “placebos” are neutral, since it cannot be excluded that the placebo itself might have included something which led to physiologic effects and detected changes.

In the study by Allen et al. [32] both dark chocolate and dark chocolate with canola sterols decreased blood pressure when the diet was based on the AHA diet (The American Heart Association’s Diet). It was concluded that the adoption of AHA diet could have led to modest reductions in blood pressure during the course of the study. In our study, the participants were recruited based on their mild hypertension. Twelve of the 22 participants’ status improved from hypertensive to normotensive in the course of the present study, but without a clear link to dark chocolate consumption. Similar to the findings in the study by Allen et. al. [32], the overall changes in the diet may have contributed the most to the positive trends seen in the current study. While the different energy intakes in the two periods may be regarded as a limitation in our study, the intention was to study a natural situation where individuals add chocolate to their existing diet. In order to avoid deliberate positive energy balance, the baseline energy intakes in both arms were first reduced by restricting the consumption of snacks.

Flow-mediated dilation (FMD) of the peripheral conduit arteries is one of the most widely used tests of endothelial function. CAVI is a new method which reflects the stiffness of the whole arterial segment, and it correlates with FMD [33]. CAVI changes over a short period of time in response to alterations in circulatory conditions; it is sensitive to subclinical changes in major arteries before impairment and it can be a reliable indicator of treatment effectiveness or modification in lifestyle [34]. Healthy lifestyle, physical exercise and smoking cessation, for example, prevent and treat early vascular aging [35, 36].

In contrast to results from our study, consumption of dark chocolate has had a beneficial effect on vascular function by improving endothelial function, measured by flow mediated dilatation both in short-terms (i.e., 2 wks) interventions [17, 37] and in acute-response studies [38, 39] without affecting arterial stiffness [39]. The duration of these studies was considerably shorter in comparison to ours. The quantity of daily dark chocolate consumption in the aforementioned studies was similar to, or substantially more (46 or 100 g/day) than the 49 g/day dose in the current study.

In the one-year intervention study of Curtis et al. [40] conducted in postmenopausal Type 2 Diabetes patients, dark chocolate consumption had a positive effect on arterial stiffness in a sub-group analysis of 35 participants. In a cross-over study where healthy participants were assigned to receive five treatments of daily intake of cocoa power with different doses of flavanols for one week each [41], cocoa consumption improved FMD and arterial stiffness. In our study, the amount of flavanols from dark chocolate was smaller than in the above-mentioned studies. Moreover, the power calculation was based on blood pressure as the main outcome, not arterial stiffness. The length of our intervention might also have been too short to achieve significant changes in arterial stiffness. In the study of Curtis et al. [40], the participants were diabetic women whereas in our study there were more men than women, and the participants were otherwise healthy, except mild hypertension.

Previous studies on dark chocolate and glucose metabolism have shown positive or neutral effects [28, 40]. In the present study, we found no effects of added dark chocolate on glucose or insulin, during the reduced snack consumption intervention. Even though the exact mechanisms are not well understood, it has been demonstrated that the acute ingestion of dark chocolate [42] or the addition of cocoa to various foods [43] can stimulate insulin responses during the post-prandial period. Moreover, the consumption of dark chocolate has induced positive effects on insulin resistance and sensitivity in short-term (i.e., 2 to 4 weeks) studies [16, 17, 28, 30] and in one study lasting one year [40].

Dark chocolate contains saturated fatty acids which have shown to be atherogenic. However, the overall atherogenic effect of a product (chocolate, in this case) is modulated by many other ingredients, not only saturated fatty acids. For instance, chocolate also contains linoleic and oleic acids, known to modulate cholesterol metabolism in a health-promoting way. Despite its saturated fatty acid content, dark chocolate has lowered both total- and LDL cholesterol in previous studies [11, 17, 30, 32] or has lowered one or the other [40, 44]. Consumption of dark chocolate has increased HDL cholesterol concentrations both in short- and long-term studies including healthy and Type 2 Diabetes patients [13, 26, 45–47].

In most studies, consumption of dark chocolate has not had any impact on triglycerides, with the exception of two studies where acute consumption increased triglyceride concentration [48] and an improvement occurred when dark chocolate and almonds were consumed as part of a low-fat diet [49]. The amount of dark chocolate cannot explain the results obtained from studies mentioned above, since doses were very different among them, and a dose–response cannot be found. The amount of dark chocolate consumed, namely 44–45 g/day, and study duration (8 wks), were comparable to our intervention in two studies [32, 45]. Improvement of cholesterol concentrations occurred even after consumption of one third [47] or half [40] of the amount of dark chocolate, compared to the present intervention. Diet modification and energy-restriction could have affected the observed changes [32, 47]. In our study, however, neither snack-restriction nor dark chocolate consumption seemed to have an effect on lipid concentrations.

Proportion of body fat and total fat mass tended to decrease during the study periods, but the change was not significant. The result of the present study is similar to previous studies [50, 51] which showed improvements in anthropometrics with a energy-reduced diet including a daily dark chocolate snack without or with the cocoa beverage, respectively. In both studies the intervention period was 18 weeks and the participants were overweight/obese women. In our study, snack restriction induced a small weight loss and added consumption of dark chocolate apparently prevented the loss. Also, in the study of Sarria et al. [52], regular consumption of a cocoa product rich in fibre for 4 wks showed a slight decrease in body fat but no change in weight.

Several intervention studies have shown that consumption of dark chocolate may have beneficial effects on cardiovascular health [7, 53]. Even if it would be tempting to recommend dark chocolate as a daily snack, long-term intake of chocolate could in theory have a negative effect on the diet. Intake of chocolate may increase the proportion of fat in the diet, and this consequently may increase energy density and decrease nutrient density. We did not find any significant differences in nutrient intakes during the study periods. Surprisingly, we did not even see a decrease in the energy intake during the control (no chocolate) period. One limitation of the study was self-reported diaries which might have given inaccurate data. The number of participants was too small to validate the actual intake during the intervention. However, calcium intake during the control period was significantly higher than during the chocolate intervention. Dairy products are usually consumed as a snack, and during the control period the participants may still have consumed more of these products. Although calcium may, in theory, have a blood pressure-lowering effect [54], the intakes in general were so high in our study that it is very unlikely the small difference had any practical significance on blood pressure.

## Conclusions

In this study, the reduced snack consumption and inclusion of 49 g dark chocolate daily as part of a diet of mildy hypertensive participants had no significant effects on cardiovascular risk factors during 8 wks. Perhaps the positive effects were already caused by the decreased snack intake and therefore, the addition of dark chocolate failed to produce beneficial effects. However, apart from a small effect on body weight (dark chocolate seemingly prevented a slight decrease in body weight during the control period), no other negative effects were observed.

## Abbreviations

BMI: Body mass index
CVD: Cardiovascular disease
FMD: Flow-mediated dilation
SBP: Systolic blood pressure
DBP: Diastolic blood pressure
CAVI: Cardio-ankle vascular index
HDL: High density lipoprotein
LDL: Low density lipoprotein
GLU: Serum glucose
INS: Serum insulin
HOMA: Homeostasis model assessment of insulin resistance
AUC: Areas under the response curve

## References

[1] Crozier A, Jaganath IB, Marks S, Saltmarsh M, Clifford MN, Crozier A, Ashihara H (2006). Secondary metabolites as dietary components in plant-based foods and beverages. Plant secondary metabolites: occurence, structure and role in the human health.

[2] Hooper L, Kroon PA, Rimm EB, Cohn JS, Harvey I, Le Cornu KA (2008). Flavonoids, flavonoid-rich foods, and cardiovascular risk: a meta-analysis of randomized controlled trials. Am J Clin Nutr.

[3] Chong MF, Macdonald R, Lovegrove JA (2010). Fruit polyphenols and CVD risk: a review of human intervention studies. Br J Nutr.

[4] Williamson G, Manach C (2005). Bioavailability and bioefficacy of polyphenols in humans. II. Review of 93 intervention studies. Am J Clin Nutr.

[5] Lee KW, Kim YJ, Lee HJ, Lee CY (2003). Cocoa has more phenolic phytochemicals and a higher antioxidant capacity than teas and red wine. J Agric Food Chem.

[6] Hammerstone JF, Lazarus SA, Mitchell AE, Rucker R, Schmitz HH (1999). Identification of procyanidins in cocoa (Theobroma cacao) and chocolate using high-performance liquid chromatography/mass spectrometry. J Agric Food Chem.

[7] Shrime MG, Bauer SR, McDonald AC, Chowdhury NH, Coltart CE, Ding EL (2011). Flavonoid-rich cocoa consumption affects multiple cardiovascular risk factors in a meta-analysis of short-term studies. J Nutr.

[8] Buijsse B, Feskens EM, Kok FJ, Kromhout D (2006). Cocoa intake, blood pressure, and cardiovascular mortality: The zutphen elderly study. Arch Intern Med.

[9] Djousse L, Hopkins PN, North KE, Pankow JS, Arnett DK, Ellison RC (2011). Chocolate consumption is inversely associated with prevalent coronary heart disease: the National Heart, Lung, and Blood Institute Family Heart Study. Clin Nutr.

[10] Petrone AB, Gaziano JM, Djoussé L (2014). Chocolate consumption and risk of heart failure in the Physicians’ Health Study. Eur J Heart Fail.

[11] Grassi D, Desideri G, Necozione S, Lippi C, Casale R, Properzi G (2008). Blood pressure is reduced and insulin sensitivity increased in glucose-intolerant, hypertensive subjects after 15 days of consuming high-polyphenol dark chocolate. J Nutr.

[12] Desch S, Kobler D, Schmidt J, Sonnabend M, Adams V, Sareban M (2010). Low vs. higher-dose dark chocolate and blood pressure in cardiovascular high-risk patients. Am J Hypertens.

[13] Hamed MS, Gambert S, Bliden KP, Bailon O, Singla A, Antonino MJ (2008). Dark chocolate effect on platelet activity, C-reactive protein and lipid profile: a pilot study. South Med J.

[14] Sudano I, Flammer AJ, Roas S, Enseleit F, Ruschitzka F, Corti R (2012). Cocoa, blood pressure, and vascular function. Curr Hypertens Rep.

[15] Lilamand M, Kelaiditi E, Guyonnet S, Antonelli Incalzi R, Raynaud-Simon A, Vellas B (2014). Flavonoids and arterial stiffness: promising perspectives. Nutr Metab Cardiovasc Dis.

[16] Grassi D, Lippi C, Necozione S, Desideri G, Ferri C (2005). Short-term administration of dark chocolate is followed by a significant increase in insulin sensitivity and a decrease in blood pressure in healthy persons. Am J Clin Nutr.

[17] Grassi D, Necozione S, Lippi C, Croce G, Valeri L, Pasqualetti P (2005). Cocoa reduces blood pressure and insulin resistance and improves endothelium-dependent vasodilation in hypertensives. Hypertension.

[18] Taubert D, Berkels R, Roesen R, Klaus W (2003). Chocolate and blood pressure in elderly individuals with isolated systolic hypertension. JAMA.

[19] Robbins RJ, Leonczak J, Li J, Johnson JC, Collins T, Kwik-Uribe C (2013). Flavanol and procyanidin content (by Degree of Polymerization 1–10) of chocolate, cocoa liquors, cocoa powders, and cocoa extracts: first action 2012.24. J AOAC Int.

[20] Shirai K, Utino J, Saiki A, Endo K, Ohira M, Nagayama D (2013). Evaluation of blood pressure control using a new arterial stiffness parameter, cardio-ankle vascular index (CAVI). Curr Hypertens Rev.

[21] Schwartz MW, Boyko EJ, Kahn SE, Ravussin E, Bogardus C (1995). Reduced insulin secretion: an independent predictor of body weight gain. J Clin Endocrinol Metab.

[22] Matthews DR, Hosker JP, Rudenski AS, Naylor BA, Treacher DF, Turner RC (1985). Homeostasis model assessment: insulin resistance and β-cell function from fasting plasma glucose and insulin concentrations in man. Diabetologia.

[23] Ried K, Sullivan TR, Fakler P, Frank OR, Stocks NP (2012). Effect of cocoa on blood pressure. Cochrane Database Syst Rev.

[24] Almoosawi S, Fyfe L, Ho C, Al-Dujaili E (2010). The effect of polyphenol-rich dark chocolate on fasting capillary whole blood glucose, total cholesterol, blood pressure and glucocorticoids in healthy overweight and obese subjects. Br J Nutr.

[25] Pereira T, Maldonado J, Laranjeiro M, Coutinho R, Cardoso E, Andrade I (2014). Central arterial hemodynamic effects of dark chocolate ingestion in young healthy people: a randomized and controlled trial. Cardiol Res Pract.

[26] Taub PR, Ramirez-Sanchez I, Ciaraldi TP, Perkins G, Murphy AN, Naviaux R (2012). Alterations in skeletal muscle indicators of mitochondrial structure and biogenesis in patients with type 2 diabetes and heart failure: effects of epicatechin rich cocoa. Clin Transl Sci.

[27] Ried K, Frank OR, Stocks NP (2009). Dark chocolate or tomato extract for prehypertension: a randomised controlled trial. BMC Complement Altern Med.

[28] Almoosawi S, Tsang C, Ostertag LM, Fyfe L, Al-Dujaili EA (2012). Differential effect of polyphenol-rich dark chocolate on biomarkers of glucose metabolism and cardiovascular risk factors in healthy, overweight and obese subjects: a randomized clinical trial. Food Funct.

[29] Faridi Z, Njike VY, Dutta S, Ali A, Katz DL (2008). Acute dark chocolate and cocoa ingestion and endothelial function: a randomized controlled crossover trial. Am J Clin Nutr.

[30] Fraga CG, Actis-Goretta L, Ottaviani JI, Carrasquedo F, Lotito SB, Lazarus S (2005). Regular consumption of a flavanol-rich chocolate can improve oxidant stress in young soccer players. Clin Dev Immunol.

[31] Taubert D, Roesen R, Lehmann C, Jung N, Schomig E (2007). Effects of low habitual cocoa intake on blood pressure and bioactive nitric oxide: a randomized controlled trial. JAMA.

[32] Allen RR, Carson L, Kwik-Uribe C, Evans EM, Erdman JW (2008). Daily consumption of a dark chocolate containing flavanols and added sterol esters affects cardiovascular risk factors in a normotensive population with elevated cholesterol. J Nutr.

[33] Endo K, Saiki A, Ohira M, Miyashita Y, Shirai K (2011). Cardio-ankle vascular index may reflect endothelial function in type 2 diabetes. Int J Clin Pract.

[34] Sun CK (2013). Cardio-ankle vascular index (CAVI) as an indicator of arterial stiffness. Integr Blood Press Control.

[35] Maeda S, Zempo-Miyaki A, Sasai H, Tsujimoto T, So R, Tanaka K (2015). Lifestyle modification decreases arterial stiffness in overweight and obese Men: dietary modification vs. Exercise training. Int J Sport Nutr Exerc Metab.

[36] Noike H, Nakamura K, Sugiyama Y, Iizuka T, Shimizu K, Takahashi M (2010). Changes in cardio-ankle vascular index in smoking cessation. J Atheroscler Thromb.

[37] Engler MB, Engler MM, Chen CY, Malloy MJ, Browne A, Chiu EY (2004). Flavonoid-rich dark chocolate improves endothelial function and increases plasma epicatechin concentrations in healthy adults. J Am Coll Nutr.

[38] Hermann F, Spieker LE, Ruschitzka F, Sudano I, Hermann M, Binggeli C (2006). Dark chocolate improves endothelial and platelet function. Heart.

[39] Vlachopoulos C, Aznaouridis K, Alexopoulos N, Economou E, Andreadou I, Stefanadis C (2005). Effect of dark chocolate on arterial function in healthy individuals. Am J Hypertens.

[40] Curtis PJ, Sampson M, Potter J, Dhatariya K, Kroon PA, Cassidy A (2012). Chronic ingestion of flavan-3-ols and isoflavones improves insulin sensitivity and lipoprotein status and attenuates estimated 10-year CVD risk in medicated postmenopausal women with type 2 diabetes: a 1-year, double-blind, randomized, controlled trial. Diabetes Care.

[41] Grassi D, Desideri G, Necozione S, di Giosia P, Barnabei R, Allegaert L (2015). Cocoa consumption dose-dependently improves flow-mediated dilation and arterial stiffness decreasing blood pressure in healthy individuals. J Hypertens.

[42] Davison G, Callister R, Williamson G, Cooper KA, Gleeson M (2012). The effect of acute pre-exercise dark chocolate consumption on plasma antioxidant status, oxidative stress and immunoendocrine responses to prolonged exercise. Eur J Nutr.

[43] Brand-Miller J, Holt SHA, de Jong V, Petocz P (2003). Cocoa powder increases postprandial insulinemia in lean young adults. J Nutr.

[44] Polagruto JA, Wang-Polagruto JF, Braun MM, Lee L, Kwik-Uribe C, Keen CL (2006). Cocoa flavanol-enriched snack bars containing phytosterols effectively lower total and low-density lipoprotein cholesterol levels. J Am Diet Assoc.

[45] Mellor DD, Sathyapalan T, Kilpatrick ES, Beckett S, Atkin SL (2010). High-cocoa polyphenol-rich chocolate improves HDL cholesterol in Type 2 diabetes patients. Diabet Med.

[46] Mursu J, Voutilainen S, Nurmi T, Rissanen TH, Virtanen JK, Kaikkonen J (2004). Dark chocolate consumption increases HDL cholesterol concentration and chocolate fatty acids may inhibit lipid peroxidation in healthy humans. Free Radic Biol Med.

[47] Wan Y, Vinson JA, Etherton TD, Proch J, Lazarus SA, Kris-Etherton PM (2001). Effects of cocoa powder and dark chocolate on LDL oxidative susceptibility and prostaglandin concentrations in humans. Am J Clin Nutr.

[48] Lettieri-Barbato D, Villaño D, Beheydt B, Guadagni F, Trogh I, Serafini M (2012). Effect of ingestion of dark chocolates with similar lipid composition and different cocoa content on antioxidant and lipid status in healthy humans. Food Chem.

[49] Kurlandsky SB, Stote KS (2006). Cardioprotective effects of chocolate and almond consumption in healthy women. Nutr Res.

[50] Piehowski KE, Preston AG, Miller DL, Nickols-Richardson SM (2011). A reduced-calorie dietary pattern including a daily sweet snack promotes body weight reduction and body composition improvements in premenopausal women who are overweight and obese: a pilot study. J Am Diet Assoc.

[51] Nickols-Richardson SM, Piehowski KE, Metzgar CJ, Miller DL, Preston AG (2014). Changes in body weight, blood pressure and selected metabolic biomarkers with an energy-restricted diet including twice daily sweet snacks and once daily sugar-free beverage. Nutr Res Pract.

[52] Sarria B, Martinez-Lopez S, Sierra-Cinos JL, Garcia-Diz L, Mateos R, Bravo L (2014). Regular consumption of a cocoa product improves the cardiometabolic profile in healthy and moderately hypercholesterolaemic adults. Br J Nutr.

[53] Hooper L, Kay C, Abdelhamid A, Kroon PA, Cohn JS, Rimm EB (2012). Effects of chocolate, cocoa, and flavan-3-ols on cardiovascular health: a systematic review and meta-analysis of randomized trials. Am J Clin Nutr.

[54] da Silva Ferreira T, Torres MR, Sanjuliani AF (2013). Dietary calcium intake is associated with adiposity, metabolic profile, inflammatory state and blood pressure, but not with erythrocyte intracellular calcium and endothelial function in healthy pre-menopausal women. Br J Nutr.

